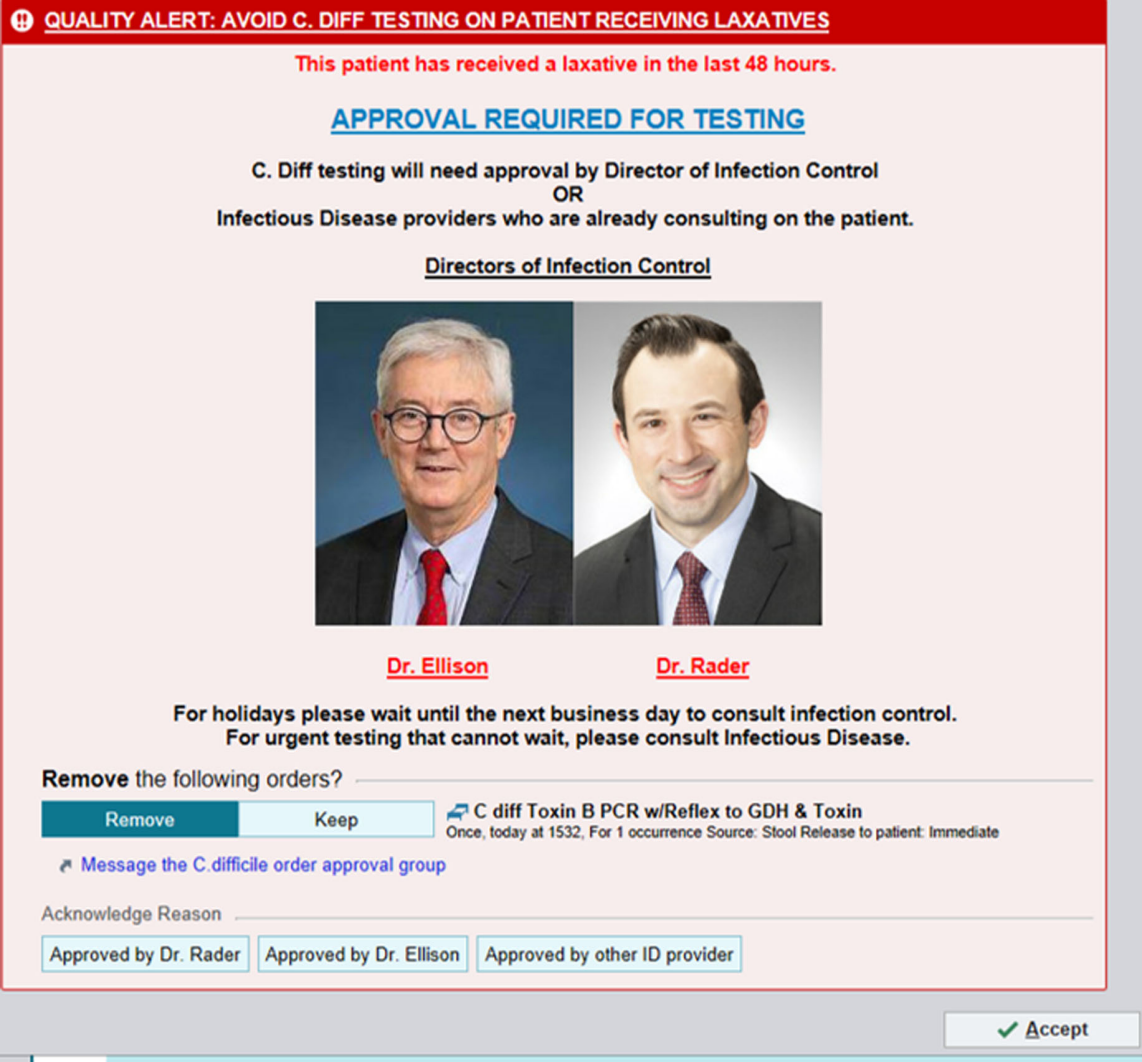# 271 Influenza Positivity in Acute Myocardial Infarction Compared With Other Emergency Department Diagnoses

**DOI:** 10.1017/ash.2026.10634

**Published:** 2026-06-23

**Authors:** Theodore (Ted) Rader, Prince Philip, Debbie Mack, Richard Ellison

**Affiliations:** 1 UMass Memorial Medical Center; 2 Ummmc; 3 University of Massachusetts Medical School

## Abstract

**Background:** Clostridioides difficile infection (CDI) is a leading cause of healthcare-associated infection in the United States. Diagnostic stewardship is a core element of reducing inappropriate CDI testing. **Methods:** In response to rising rates of hospital onset CDI (HO-CDI), our institution implemented a “hard-stop” clinical practice alert (OPA) in the Electronic Medical Record (EMR) in July 2024, shown in Figure 1. The OPA fired when a CDI test was ordered on patients hospitalized greater than 48 hours who had received laxatives within the previous 48 hours or who had a positive test within the previous 7 days and was active from 7AM-5PM Monday through Friday. OPA completion to order CDI testing required approval from either the Infection Prevention & Control Medical Directors (IPCMD) or a consulting Infectious Disease (ID) physician if patient was followed by ID. To facilitate communication, a dedicated “chat group” was created in the EMR instant messaging to specifically respond to a request for testing by the IPCMDs and a link to the chat was included in the OPA. EMR documentation of the approving IPCMD was required, and logs were regularly reviewed for compliance. Every HO-CDI case at our institution undergoes a multidisciplinary review with the staff and primary providers involved in the care of the patient at time of diagnosis to identify a “driver” for the case. Testing Stewardship, defined as inappropriate testing due to noncompliance with institutional CDI testing guidance, is one of the 5 drivers that can be assigned during this review process. **Results:** Over the 12.5 months following implementation the OPA was triggered 432 times on 214 unique patients with testing performed 79 times (0.18 tests per trigger). Twenty of 79 tests (25%) were positive for toxigenic C. difficile. In the 12 months prior to the OPA, there were 17 of 88 HO-CDI cases attributed to Testing Stewardship compared to 4 of 58 in the 12 months after the OPA (18/80 vs 4/58 by fiscal year). In the context of multiple interventions, NHSN-defined HO-CDI declined from 68 in FY 2023, 80 in FY 2024 to 58 in FY2025, corresponding to rates of 2.22, 3.13 and 1.7 per 10,000 patient days, respectively. **Conclusion:** A multicomponent OPA requiring approval for CDI testing for specific parameters was successfully implemented. This intervention contributed to improved diagnostic stewardship and reduction on HO-CDC cases Figure 1: C. difficile Hard Stop OPA example for patient with recent laxative administration

**Figure:**